# The third complete chloroplast genome of *Melicope pteleifolia* (Rutaceae): a widely used folk medicinal herb

**DOI:** 10.1080/23802359.2021.1903364

**Published:** 2021-03-24

**Authors:** Huihui Liu, Ziyuan Chen, Yuyang Zhao, Junhui Zhou, Qing Ma, Xinhong Wang, Zhongyi Hua

**Affiliations:** a China Resources Sanjiu Medical & Pharmaceutical Co, Ltd, Shenzhen, PR China; b National Resource Center for Chinese Materia Medica, China Academy of Chinese Medical Sciences, Beijing, PR China

**Keywords:** Chloroplast genome, *Melicope pteleifolia*, genomic resources

## Abstract

*Melicope pteleifolia* commonly known as thin evodia, is an herb used to therapy eczema, dermatitis, and other ailments in traditional Chinese medicine. Here, we reported the third complete chloroplast genome of *M. pteleifolia* based on next-generation sequencing. The third chloroplast genome of *M. pteleifolia* is 158,933 bp in length consisting of large and small single-copy regions of length 85,020 and 18,607 bp, separated by two IR regions of 27,683 bp. The overall GC content was 38.30%. De novo assembly and annotation showed the chloroplast genome of *M. pteleifolia* encodes 134 genes, including 89 protein-coding genes, 37 *tRNA* genes, and eight *rRNA* genes. A huge intraspecies variation was found with 248 SNPs and 97 INDELs among three assemblies of *M. pteleifolia*. Phylogenetic tree indicated that three assemblies of *M. pteleifolia* form a clade, sister to the genus *Phellodendron* and *Casimiroa.*

*Melicope pteleifolia* (Rutaceae) is a shrub commonly distributed in south of China and used as a traditional Chinese medicine to treat eczema, dermatitis, rheumatic arthralgia, and other ailments (Flora of China Committee 2008; Yao et al. 2020). Modern pharmacological studies reported that its crude extracts exhibited analgesic, anti-inflammatory, anti-tumor, and antioxidative effects (Shaari et al. 2011; Nguyen et al. 2016; Kabir et al. 2018; Lee et al. 2019). So, it is worth doing some works to utilize *M. pteleifolia* better, including distinct *M. pteleifolia* from its closely related species and investigate its intraspecies variation to ensure the safety of usage. However, very less is known about the genomics information of *M. pteleifolia,* even the genus *Melicope*. Up to now, chloroplast genomes from about 26 species of Rutaceae have been sequenced and published, and two of them belongs to the *M. pteleifolia*. To evaluate the intraspecies variation, we sequenced the third chloroplast genome of a *M. pteleifolia* individual growing in wild field in the Jingxi, Guangxi province (105°58′E, 23°06′N), 780 km away from the first individual isolated (Yu et al. 2021). The relationship between *M. pteleifolia* and other Rutaceae species was analyzed in this article with hope to provide better understanding of the phylogenetic status of *Melicope* and *M. pteleifolia*.

We collected fresh healthy leaves from *M. pteleifolia* species growing in the Jingxid, Guangxi province (105°58′E, 23°06′N). Voucher specimen was stored in herbarium of Institute of Chinese Materia Medica (CMMI, accession number 451025LY0636), China Academy of Chinese Medical Sciences. The DNA extraction and sequencing were performed as described before (Liu et al. 2020). Briefly, the sequencing library was constructed using NEB Next^®^ Ultra DNA Library Prep Kit for Illumina^®^ (NEB, Ipswich, MA). Paired-end (2 × 150 bp) sequencing was performed by Novogene Bioinformatics Technology Co. Ltd (Beijing, China), using the Illumina Hiseq X-Ten platform. About 5.0 Gb of sequence data was obtained after sequencing and base quality control. The paired-end reads were then assembled with GetOrganelle (Jin et al. 2020) based on the default reference sequences. The complete genome sequence was annotated by both GeSeq (Tillich et al. 2017) and PGA (Qu et al. 2019) based on previously reported *Amborella trichopoda* chloroplast genome (NC_005086.1) and *Citrus reticulata* chloroplast genome (NC_034671.1). Finally, we checked and merged the annotation from GeSeq and PGA manually. The annotated genomic sequence had been submitted to GenBank with the accession number MW263046.

The chloroplast genome of *M. pteleifolia* is 158,933 bp in length consisting of large and small single-copy regions of length 85,020 and 18,607 bp, separated by two IR regions of 27,683 bp. GC content was 38.30%. The genome consisted of 134 different genes, including 89 protein-coding genes, 37 distinct *tRNA* genes, and eight *rRNA* genes.

Based on the alignment of the three chloroplasts of *M. pteleifolia*, there is significant difference between our sequence and the published sequences. 247 single nucleotide polymorphisms (SNPs) and 96 insertions and deletions (INDELs) were identified between our sequence and MW046256. 121 SNPs and 26 INDELs were found in CDS region. Number of intraspecific variations identified between our sequence and NC_050882 is large compared to variations between NC_050882 and MW046256 (4 SNPs and 7 INDELs) or other species (Park et al. 2019; Park and Oh 2020), indicating high-level genetic diversity exists in *M. pteleifolia*. Results in this study suggested that the previous samples (NC_050882 and MW046256) may come from the same population, but our sample collected in Guangxi may come from a different population.

To confirm the phylogenetic location of *M. pteleifolia* within the family of Rutaceae, a total of 28 complete cp genomes of Rutaceae (including two previous *M. pteleifolia* chloroplast assemblies) were obtained from GenBank, and *Azadirachta indica* in Meliaceae family was used as out-group. The 30 complete chloroplast sequences were aligned by the MAFFT version 7 software (Katoh and Standley 2013). Phylogenetic analysis was conducted based on maximum likelihood (ML) analyses implemented in IQ-TREE version 2.0.5 (Minh et al. 2020) under the TVM + F + R2 nucleotide substitution model, which was selected by ModelFinder (Kalyaanamoorthy et al. 2017). Support for the inferred ML tree was inferred by bootstrapping with 1000 replicates. Phylogenetic analysis results strongly supported that *M. pteleifolia* was closely related to the genus *Phellodendron* and *Casimiroa* (Figure 1).

**Figure 1.**
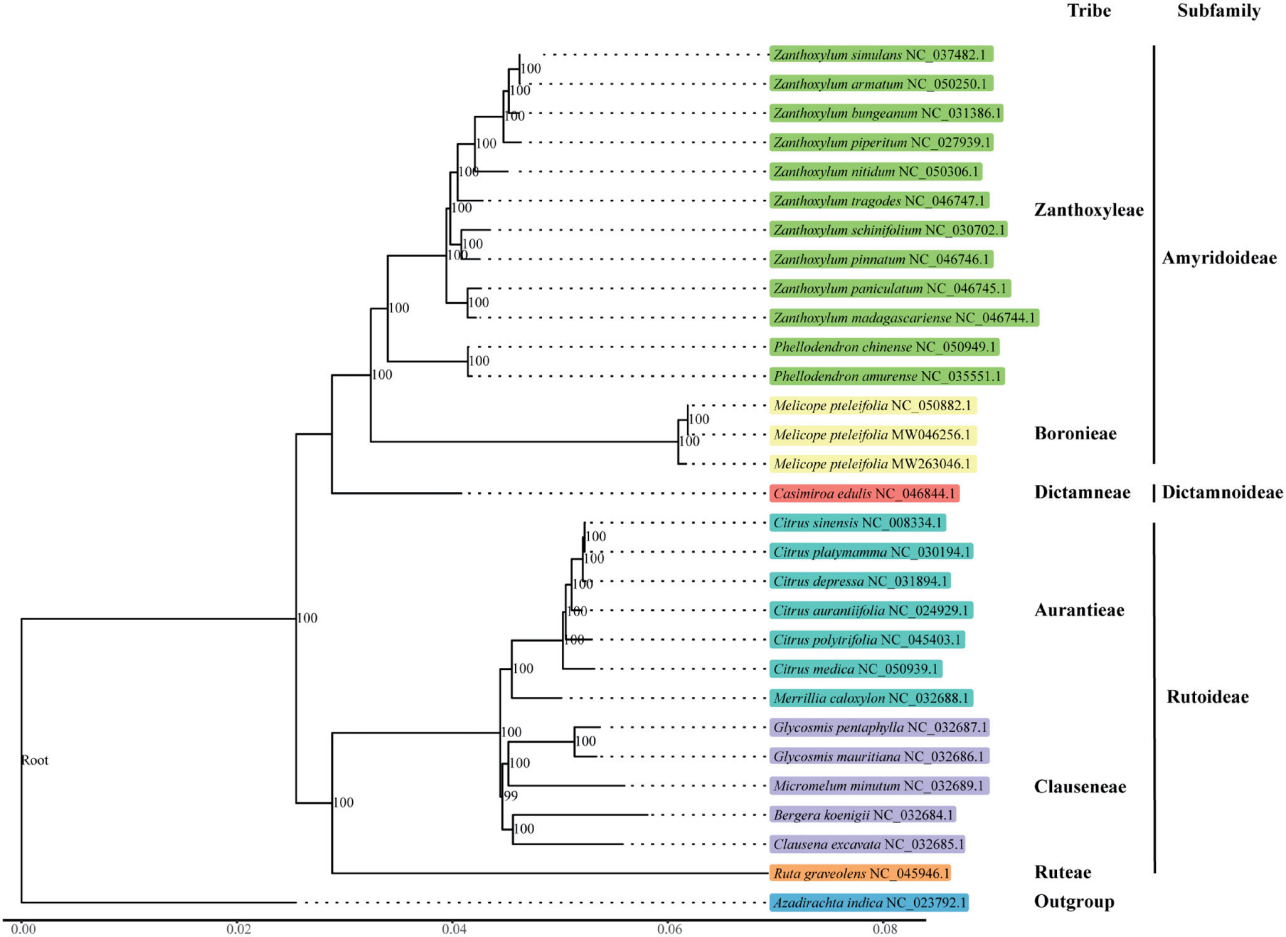
Phylogenetic tree reconstruction of 28 taxa using maximum likelihood (ML) methods based on the chloroplast genome sequences. Numbers in parentheses at each node are bootstrap supports (%).

## Data Availability

The data that support the findings of this study are openly available in GenBank of NCBI at https://www.ncbi.nlm.nih.gov, reference number MW263046.
